# P38 Virtual escape room game evaluation for antimicrobial stewardship (AMS) learning

**DOI:** 10.1093/jacamr/dlag102.044

**Published:** 2026-06-26

**Authors:** Naomi Fleming, Amy Chapman, Tasha Nightingale, Clarice Benney, Simi Odimayo, Amy Selby, Sumita Pai, Netta Tyler, Christianne Micallef, Sandra Satkeviciene, Sheena Patel

**Affiliations:** NHS England, UK; Health Innovation East, UK; Health Innovation East, UK; Health Innovation East, UK; Health Innovation East, UK; Health Innovation East, UK; Royal Papworth Hospital NHS Foundation Trust, Cambridge, UK; Royal Papworth Hospital NHS Foundation Trust, Cambridge, UK; Cambridgeshire and Peterborough ICB, UK; East and North Hertfordshire Teaching NHS Trust, UK; East and North Hertfordshire Teaching NHS Trust, UK

## Abstract

**Objectives:**

To: (i) Design and develop an AMS gamified learning tool for healthcare professionals (HCP). (ii) Evaluate the impact through participant surveys, to determine: (a) the effectiveness of the AMS game on knowledge improvement; (b) the acceptability of the training approach; and (c) self-reported AMS behaviour change intention (the team acknowledge that intention to change behaviour is not actual behaviour change). (iii) Understand the effectiveness of a gamified learning tool for training on AMS.

**Methods:**

A gamified learning tool was developed by AMS specialists and Health Innovation East. AMS specialists developed the clinical content, and the design and evaluative element were developed iteratively by Health Innovation East. The final design comprised escaping five rooms. Audiovisual media files introduced each stage, including a map to demonstrate progress and narrative text was enhanced through sound effects and graphics. To ensure the game was accessible, all audio-visual files were accompanied with plain text. The game was uploaded to *Zoho Survey* for digital distribution. Evaluation measures were included at the beginning and end of the game. Self-reported scores were collected using a combination of Likert scales (0-10) and free text responses. The game was promoted in October and November 2025 to the target audience HCP in the East of England. It was open between 1 and 30^th^ November 2025 to coincide with World Antimicrobial Awareness Week 18-24 November.

**Results:**

The game had 4774 visits, and 749 completed the training. The majority of participants, 46.5% (*n*=348) were pharmacists, 13.5% (*n*=101) nurses, 13.2% (*n*=99) doctor/medics, 9.75% (*n*=73) pharmacy technicians, 4.1% (*n*=31) categorized as ‘others’, 2.9% (*n*=22) advanced clinical practitioners, 2.8% (*n*=21) students, 2.4% (*n*=18) admin/managerial, 2.2% (*n*=17) microbiologists, and 1.1% (*n*=8) were allied health professionals. 1.5% (*n*=11) did not select a role. Self-reported understanding and knowledge of AMS principles were significantly improved following participation (median=7, versus median=8; Wilcoxon signed rank test, *n*=749, V=23,783.5 *P*<0.00001). Self-reported confidence in applying AMS principles following participation also significantly improved (median=7, versus median=8); Wilcoxon signed rank test, *n*=749, V=25, 320 *P*<0.00001). The learning tool demonstrated high levels of acceptability as demonstrated by qualitative feedback and scores, 40.6% (*n*=304) gave the maximum score 10: very engaging; median score=9.

**Conclusions:**

The gamified learning tool was found to be an effective way to engage HCP about AMS. The escape room concept and quiz content were positively received and demonstrated high levels of acceptability for the participants who took part. The learning tool had a positive impact on AMS awareness and demonstrated clear potential for changes in AMS behaviours by supporting prescribing clinicians to feel more confident in their application of AMS knowledge, particularly in hospital-based settings. Improvements suggested included accompanying answers with a linked resource for further learning and providing a visual representation of correct or incorrect answers e.g. ticks or crosses. The game will be reopened due to popular demand, with content verified 6 monthly.

